# Response Surface Methodology as an Experimental Strategy for Ultrasound-Assisted Extraction of Phenolic Compounds from Artichoke Heads

**DOI:** 10.3390/antiox12071360

**Published:** 2023-06-29

**Authors:** Valentina Melini, Francesca Melini, Francisco Javier Comendador

**Affiliations:** CREA Research Centre for Food and Nutrition, Via Ardeatina 546, I-00178 Roma, Italy; francesca.melini@crea.gov.it (F.M.); fjavier.comendador@crea.gov.it (F.J.C.)

**Keywords:** response surface methodology, ultrasound-assisted extraction, phenolic compounds, flavonoids, artichokes, HPLC

## Abstract

The accurate quantification of phenolic compounds (PCs) in foods has become mandatory for a reliable estimation of PCs dietary intake. However, the extraction step of these molecules from the food matrix is a challenging and complex task. To manage the current lack of an official or generally accepted procedure for the recovery of phenolics, the application of statistical and mathematical tools, such as the response surface methodology (RSM), that allow the optimization of extraction parameters and the acquisition of the best output, has become the analytical approach of choice. The aim of this study was to apply an RSM-optimized ultrasound-assisted procedure to extract phenolic compounds from artichoke (*Cynara cardunculus* L. var. *scolymus* (L.) Hegi, cultivar “Campagnano”) heads. The effect of extraction time, temperature, and solvent-to-sample ratio on the profile and content of phenolic acids and flavonoids was investigated. The total phenolic content was 488.13 ± 0.56 mg GAE 100 g^−1^ dry matter (dm) and total flavonoid content was 375.03 ± 1.49 mg CAT_eq_ 100 g^−1^ dm when the optimum extraction conditions were set. The HPLC analysis showed that caffeoylquinic acid derivatives (i.e., cynarin and 1,5-*O*-dicaffeoylquinic acid) were the main compounds in globe artichokes. Caffeic and *p*-coumaric acids were also identified. In regard to flavonoids, only the flavone luteolin-7-*O*-glucoside was identified.

## 1. Introduction

Phenolic compounds (PCs) are common secondary plant metabolites with over 8000 known structures. They are produced by plants via either the shikimic acid pathway, generating the phenyl propanoid (C_6_-C_3_) skeleton, or the acetate pathway [1], serving as a building block for polymeric two-carbon units, during normal metabolic processes or in response to environmental conditions, e.g., wounds, temperature, UV radiation, and infection. PCs comprise molecules with different chemical structures, where an aromatic ring is linked to one or more hydroxyl substituents. PCs range from simple phenolic acids to complex flavonoids [2].

This heterogenous class of compounds is found in most foods of vegetable origin. Fruit and vegetables are the main source of PCs, but whole grains and pseudocereals are rich in phytochemicals as well [3,4,5,6]. Phenol-Explorer, i.e., the comprehensive database on polyphenol content in foods, reports the content for 500 phenolic compounds in over 400 foods, for a total of 35,000 values. In addition, an increasing number of studies on the determination of phytochemicals in food has been published over the last few years.

This flourishing of studies is related to the beneficial health properties that PCs are increasingly claimed to have [2]. They are, in fact, asserted to act as antioxidants by preventing the transition metal-mediated formation of hydroxyl free radicals and by scavenging oxygen, nitrogen, and chlorine reactive species [7]. This means that the consumption of foods rich in phytochemicals can contribute to preventing some types of cancers and cardiovascular diseases [8] and to protection against the onset of neurodegenerative diseases [9]. Within this framework, the accurate quantification of phenolic compound content in foods becomes the pre-requisite for a sound and reliable estimation of PCs dietary intake.

Over the last few years, studies have increasingly pointed out that the extraction step of phytochemicals from the food matrix, in terms of extraction yield and selectivity, is a challenging task [4,5,6,10,11]. This recovery, for instance, can be tricky due to the high enzyme activity in most foods and plants [12]. Hence, the identification of the extraction process must be performed very carefully to avoid the chemical alteration of the target compounds and to assure a reliable recovery thereof.

To manage the current lack of an official or generally accepted procedure for the recovery of phenolics, the application of statistical and mathematical tools to optimize extraction parameters (e.g., extracting solvent, temperature, time, and sample-to-solvent ratio) and control the process to obtain the best output has become the analytical approach of choice.

The response surface methodology (RSM) is a mathematical and statistical tool increasingly used in process and product design, where several independent variables (factors) can potentially affect the dependent variable(s) (responses). The application of RSM to a process implies several stages: the choice of the factors, the identification of the method, the selection of a suitable model, the confirmation of model adequacy, the representation of the model by 2D contour and 3D plots, and finally optimization to attain the optimal conditions. Compared to one-factor-at-a-time approaches, RSM has the advantage of optimizing specific responses influenced by variables upon a simultaneous reduction in the number of experimental trials and any associated operational costs; in addition, any possible interaction effects between the independent variables taken into consideration by models and graphical illustrations are considered.

Phytochemical extraction has been long carried out with conventional techniques, such as maceration, Soxhlet extraction, percolation, decoction, etc. [13]. However, over recent times, advanced techniques (e.g., ultrasound- and microwave-assisted extraction; supercritical fluid extraction; pulse electric field extraction and enzyme-assisted extraction) have been increasingly investigated for application in polyphenols extraction [11]. Advanced techniques are claimed to be more efficient—lower energy consumption and higher-quality extracts have been reported [13].

To the best of our knowledge, phenolic compounds have never been extracted in artichoke (*Cynara cardunculus* L. var. *scolymus* (L.) Hegi, cultivar “Campagnano”) heads via an RSM-optimized ultrasound-assisted procedures. Since artichokes are a rich source of bioactive phenolic compounds, and Italy is the main world producer with an annual production of 376,280 tons in 2021 [14], it is important to identify the optimal extraction conditions that allow for a reliable estimation of phenolic compound content in artichokes.

The aim of this study was therefore to model and optimize phenolic compound extraction in artichoke samples via RSM. An ultrasound-assisted extraction (UAE) was performed, and the effect of extraction time, temperature, and solvent-to-sample ratio on total phenolic content (TPC) and total flavonoid content (TFC), as well as on phenolic acid and flavonoid profile, was investigated.

## 2. Materials and Methods

### 2.1. Chemicals and Reagents

Folin–Ciocalteu reagent, calcium chloride, citric acid, aluminum chloride, sodium nitrate, sodium hydroxide, and RPE methanol were purchased from Carlo Erba Reagents (Milan, Italy).

Chlorogenic acid, caffeic acid, *p*-coumaric acid, *t*-ferulic acid, 1,3-dicaffeoylquinic acid, luteolin, luteolin-7-*O*-glucoside, apigenin, and apigenin-7-*O*-glucoside were purchased from Extrasynthèse (Geney, France) and Sigma-Aldrich (St. Louis, MO, USA).

HPLC-grade solvents and water purified using a Milli-Q system (Millipore Corp., Billerica, MA, USA) were used in HPLC analysis.

### 2.2. Artichoke Heads and Sample Preparation

The artichoke (*Cynara cardunculus* L. var. *scolymus* (L.) Hegi, cultivar “Campagnano”) heads analyzed in this study were harvested in situ at their place of origin, i.e., central Italy (Abruzzo region). The heads were made available by the grower immediately upon harvest in the month of May 2022. In detail, artichoke heads were harvested, shipped to the laboratory in paper boxes within two days after harvest, and made immediately available for analysis.

Sample preparation consisted in removing external bracts, chopping the heads in four main sections, dipping them in a solution of citric acid (pH 3) to avoid browning, and finely chopping the artichoke heads for freezing and freeze-drying.

### 2.3. Experimental Design

A three-level-three-factor Box–Behnken design (BBD) was set using the Design Of Experiment (DOE) tool of the Minitab Pro 18 software (Minitab Inc., State College, PA, USA) and is reported in Table 1.

Extraction temperature (X_1_; °C), extraction time (X_2_; min), and solvent-to-sample ratio (X_3_, mL g^−1^) were set as factors. Total phenolic content (TPC) and total flavonoid content (TFC) were set as responses.

The three independent factors were investigated at three levels: 0 was the midpoint to determine the experimental error, +1 was set as the high level, and −1 was set as the low level (Table 1).

The experimental design comprised 15 experiments, as specified in Table 2. Experiments were carried out randomly, according to the experimental design. 

### 2.4. Ultrasound-Assisted Extraction of Free Phenolic Compounds

Free phenolic compounds were extracted via a two-step process, coupling traditional solid–liquid extraction and ultrasounds. Briefly, a definite amount of test sample (Table 1) was placed into a PYREX™ screw cap culture tube, and 5 mL of the extracting mixture (i.e., methanol:water 80:20 *v*/*v*) was added to the tube. The tube was vortexed for 1 min to assure adequate mixing of the sample and extracting mixture and was then placed in an ultrasound bath system Elmasonic S 100 H (Elma Schmid Bauer GmbH, Singen, Germany), operating at 37 kHz. A 2 min equilibration of the test tube with the water bath temperature was performed. The setting of the extraction conditions (i.e., times and temperatures) is specified in Table 3. During the ultrasound extraction phase, water bath temperature was controlled using a glass laboratory thermometer. Extraction was carried out tube by tube, randomly, according to the experimental design in Table 3. 

After the first ultrasound extraction step, the solid–liquid solution was kept at +4 °C for 10 min and centrifuged at 7000 rpm for 10 min to recover the supernatant. The latter was stored in a tube for future analysis. An additional volume (5 mL) of the extracting mixture (methanol:water 80:20 *v*/*v*) was added to the sample, and a second extraction step was carried out under the same conditions as the first step. 

The supernatant recovered after the second extraction step was then added to the corresponding supernatant collected after the first extraction. Pooled supernatants were filtered in a 0.42 µm filter. 

Phenolics determination occurred on pooled supernatants immediately after the extraction.

### 2.5. Total Phenolic Content Determination

Total phenolic content (TPC) was determined using the Folin–Ciocalteu reagent (FCR) assay, as reported in Sompong et al. [15] and in Melini and Melini [6]. Briefly, 120 µL of filtered extract was added to 600 µL of water-diluted FCR (1:10). After three minutes, 960 µL of sodium carbonate (75 g/L) was added to adjust the system pH to a target range of 10–10.5. Test tubes were placed in a bath at 50 °C for 10 min, and after cooling the absorbance was measured at a wavelength of 760 nm against the blank reagent. For each extract, three replicates were performed.

TPC was quantified by a calibration curve of pure gallic acid within a concentration range of 22–121 µg mL^−1^ as a standard. Data were expressed as milligrams of gallic acid equivalents (GAE) per 100 g of sample on a dry matter basis (mg GAE 100 g^−1^ dm). The coefficient of determination (R^2^) of the calibration curve was 0.9905 and the regression equation was:

y = 0.0946x − 0.0825(1)

where y = absorbance at 760 nm and x = concentration (µg mL^−1^) of gallic acid.

### 2.6. Total Flavonoid Content Determination

Total flavonoid content (TFC) was determined via colorimetric assay, according to the procedure reported in Alshikh et al. [16], with slight modifications. In detail, 250 µL of extract was mixed with 1 mL distilled water and 75 µL of 5% (*w*/*v*) sodium nitrate (NaNO_2_) was added. The tube was thus allowed to stand for 5 min until the reaction was complete. Then, 75 µL of 10% (*w*/*v*) aluminum chloride (AlCl_3_) was added to the mixture and allowed to stand for 1 min more. Finally, 0.5 mL sodium hydroxide (NaOH) 1 M and 0.6 mL distilled water were added and mixed. Tubes were left to stand for 15 min in the dark at room temperature. Absorbance was measured at a wavelength of 510 nm against the blank reagent. For each extract, three replicates were performed. A calibration curve of catechin, within a concentration range of 2–20 µg mL^−1^, was used to quantitate total flavonoids. The coefficient of determination (R^2^) of the calibration curve was 0.9974 and the regression equation was:

y = 0.0399x − 0.0155(2)

where y = absorbance at 510 nm and x = concentration (µg mL^−1^) of catechin.

Results were expressed as milligrams of catechin equivalents (CAT_equ_) per 100 g of sample on a dry matter basis (mg CAT_equ_ 100 g^−1^ dm).

### 2.7. Regression Equation and Model Validation

The experimental data were fitted to the following second-order polynomial model equation:

(3) $$Y=\beta_{0}+\sum_{i=1}^{3}\beta_{i}X_{i}+\sum_{i=1}^{3}\beta_{\mathrm{ii}}X_{i}^{2}+\sum_{i=1}^{3}\sum_{j=1}^{3}\beta_{\mathrm{ij}}X_{i}X_{j}$$

where *Y* is the response variable; *X_i_* and *X_j_* are the independent variables; *β*_0_ is the intercept regression coefficient; *β_i_* is the linear regression coefficient; *β_ii_* is the squared regression coefficient; and *β_ij_* is the cross-product coefficient (first-order interaction between *X_i_* and *X_j_*). The statistical significance (*p* < 0.05) of the process parameters was verified via analysis of variance (ANOVA), which allows the estimation of the relative contribution of each control factor to the overall response.

The statistically non-significant terms (*p* > 0.05) were excluded from the model. The quality of the fit of the polynomial model equation was expressed through the regression coefficient (R^2^), whose value must be close to 1.0 in a model that describes excellent prediction efficiency. Since model prediction effectiveness must not be supported by R^2^ only [17], R^2^ adjusted (R^2^_adj_) was also calculated. The comparison of R^2^ and R^2^_adj_ values allows for the evaluation of the number of independent variables in the experiment. The *F*-value of both the regression model and the lack of fit (LOF) were expressed at a probability (*p*) of 0.05. The response was reported graphically both as contour plots, which display the shape of the response surface, and in the three-dimensional space. 

Regarding the process optimization, the composite desirability tool, available in the Minitab Response Optimizer, was used. The optimization of the three independent variables (X_1_, X_2_, and X_3_) was thus performed by maximizing the two responses, i.e., TPC and TFC. Finally, the model was validated by performing the extraction at the optimal conditions. 

### 2.8. HPLC Analysis of Extracts Obtained at Optimal Extraction Conditions

The HPLC analysis of the phenolic compounds and flavonoids was performed using a Varian ProStar HPLC system (Varian Inc., 2700 Mitchell Drive, Walnut Creek, CA, USA), equipped with a UV–Vis detector. The separation was carried out using an Inertsil^®^ ODS-3 reversed-phase column (250 × 4.6 mm, 5 μm). The elution was obtained using a gradient of water acidified with acetic acid 2.5% (Solvent A) and acetonitrile (Solvent B). The total runtime of the method was 48 min, and the concentration gradient was varied as follows: 5% B and 95% A at 0 min, 10% B and 90% A at 5 min, 10% B and 90% A at 10 min, 20% B and 80% A at 20 min, 30% B and 70% A at 30 min, 50% B and 50% A at 40–45 min, and 5% B and 95% A at 48 min. 

A constant flow rate of 1 mL/min and a temperature of 40 °C were used. All the extracts were filtered through 0.22 µm membranes, and the mobile phase was degassed before injection onto HPLC. Following the analysis of the UV–Vis spectra of the targeted individual phenolic standards, chromatograms were recorded at two wavelengths (260 and 320 nm). External standard calibration was used for phenolics and flavonoids quantification (Table 2).

### 2.9. Statistical Analysis

Statistical analyses (e.g., ANOVA, response surface methodology) were performed via: (i) Minitab Pro 18 (Minitab Inc., State College, PA, USA), which was used to set the experimental design and elaborate data; (ii) Design Expert software (version 10, Stat-Ease, Inc., Minneapolis, MN, USA), through which contour plots and surface 3D graphs were sketched; and (iii) Microsoft® Excel® for Windows 365 (version 2103) to process the data obtained from experiments.

## 3. Results and Discussion

### 3.1. Optimization of Phenolic Compound Extraction via RSM

The mean values (*n* = 3) of TPC and TFC obtained for the artichoke head extracts are presented in Table 3 and discussed in the following paragraphs.

The response of TPC ranged from 347.16 ± 15.28 to 493.88 ± 14.12 mg GAE 100 g^−1^ dm. The lowest TPC value was obtained in run #2, that is, when a total amount of 0.143 g sample (SSR = 35 mL g^−1^) was extracted at 20 °C for 10 min. The highest value was observed in run #4, when a total amount of 0.100 g (SSR = 50 mL g^−1^) sample was extracted at 40 °C for 10 min (Table 3). 

As regards TFC, the values obtained in the 15 runs ranged between 269.82 ± 15.98 (run #9) and 390.86 ± 20.78 (run #15) mg CAT_equ_ 100 g^−1^ dm. The lowest value was observed when 0.250 g (SSR = 20 mL g^−1^) was extracted for 30 min at 40 °C, while the highest was observed when 0.100 g was extracted for 20 min at 60 °C (Table 3). 

The data obtained from the BBD were fitted to the second-order polynomial equation specified above, and the model coefficient significances were estimated via analysis of variance (ANOVA; Table 4). The significant factors were ranked based on the F-value or *p*-value (probability value) with a 95% confidence level.

In terms of the regression model for TPC, the model F-value of 13.83 implies that the model is significant (*p* < 0.05). There is, in fact, only a 0.05% chance that an F-value this large could occur due to noise (Table 4). Since values of “Prob > F” less than 0.05 indicate that model terms are significant, it was observed that X_1_, X_3_, and X_1_X_3_ were significant model terms. As regards the LOF F-value, the obtained value (1.69) implies that the LOF was not significant and relative to the pure error. There is a 79.04% chance that a LOF value this large could occur due to noise.

The final empirical model in terms of coded factors, to be used to make predictions about the response for given levels of each factor, is shown in the following equation:

(4) $$Y=408.73+29.46X_{1}+37.82X_{3}+30.71X_{1}X_{3}$$

The comparison between the F value of the regression coefficients determined by Fisher’s F-test and the tabulated coefficients (F_regression_ = 13.83 > F_tabulated (9,11,0.05)_ = 3.59) showed that the first was higher, with *p* < 0.05. This shows that the model’s independent variables significantly affect the response. Furthermore, the ratio of the mean square (MS) of LOF and pure error is lower than the tabulated value (F_lack-of-fit_ = 1.69 < F_tabulated(9,2,0.05)_ = 19.385), and the insignificant LOF *p*-value (*p* > 0.05) implies that the model is valid.

As regards the total flavonoid content (TFC) response, the ANOVA for the response surface showed that this model is also significant (model F-value = 15.04), and there is only a 0.03% of chance that an F-value this large occurred due to noise (Table 4). For TFC, ANOVA showed that the solvent-to-sample ratio was the only significant term of the model (*p* < 0.05). The LOF F-value (equal to 1.08) implies that LOF is not significant relative to the pure error. There is a 56.78% chance that a LOF F-value this large could occur due to noise. ANOVA also showed a good positive correlation (R^2^ = 0.8040) between the response TFC and the significant extraction parameter. The model can thus be used to navigate the design space. 

Equation (5) is the final equation for TFC in terms of coded factors and shows the mathematical model describing the relationship between the significant independent variables and the response variable:

(5) $$Y=313.41+11.20X_{1}+37.14X_{3}+18.80X_{1}^{2}$$

Fisher’s F-test was used to confirm the statistical significance of the regression equation for TFC. It emerged that the F-value of regression coefficients was higher than the tabulated value (F_regression_ = 15.04 > F_tabulated (3,11,0.05)_ = 3.59) and the corresponding *p*-value was smaller than 0.05. This shows that the model’s independent variables significantly affected the TFC response. Moreover, the mean square of LOF and pure error ratio is inferior to the tabulated values (F_lack-of-fit_ = 1.08 < F_tabulated (9,2,0.05)_ = 19.385), and the LOF *p*-value (*p* > 0.05) shows that the model is valid.

Response surfaces were also produced to determine the optimum level of each variable for maximum productivity response. The graphical representation is a useful method to visualize the relationship between the response and the experimental level of variables.

**Figure 1:**
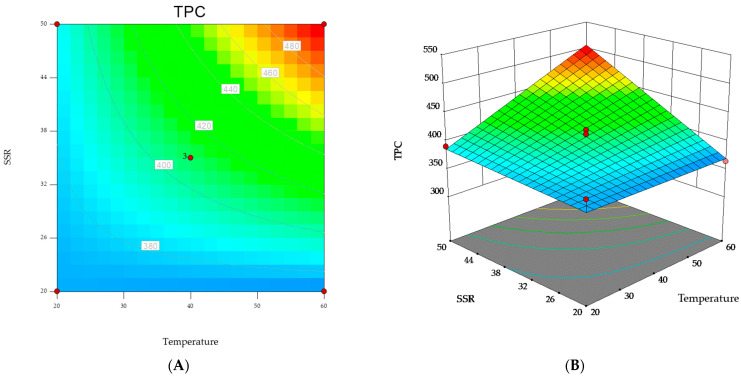
Response surfaces of TPC as a function of the interactions between extraction temperature and solvent-to-sample ratio. (**A**) contour plot; (**B**) 3D plot.

Figure 1 shows the contour plot and the 3D response surface for TPC, as a function of the interactions between the two significant variables, i.e., extraction temperature (X_2_) and sample-to-solvent ratio (X_3_). In the panels (Figure 1A,B), the non-plotted variable was kept at its zero level.

As shown in the figure, the highest values for the response (i.e., TPC) were obtained when the extraction was carried out at 60 °C and with a solvent-to-sample ratio of 50 mL g^−1^.

**Figure 2:**
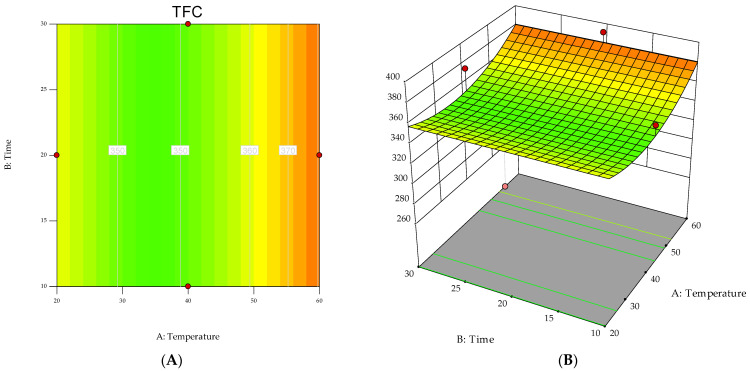
Response surfaces of TFC as a function of the interactions between extraction temperature and time. (**A**) contour plot; (**B**) 3D plot.

Figure 2 shows the contour plot and the three-dimensional response surface of the interactions between the two variables, i.e., extraction time (X_1_) and temperature (X_2_), for TFC. In the panels (A and B), the non-plotted variable was kept at its zero level. It can be observed that the highest values for the response (i.e., TFC) were obtained when the extraction was carried out at 60 °C.

### 3.2. Identification of the Optimal UAE Conditions and Validation of the Experimental Model

The application of RSM to the UAE of phenolic compounds in artichoke head samples aimed to identify the levels of the investigated experimental factors that allow maximizing the two responses, i.e., TPC and TFC.

Based on the model, maximum response of 506.72 mg GAE 100 g^−1^ dm for TPC and 380.56 mg CAT_eq_ 100 g^−1^ dm for TFC was predicted when optimal conditions (i.e., 60 °C, 20 min and 50 mL g^−1^ SSR) were applied. 

To validate the predicted model, three UAE experiments at the optimal extraction conditions were conducted, according to the extraction protocol followed for the previous experimental runs. A close match between experimental and predicted values was obtained: 488.13 ± 0.56 mg GAE 100 g^−1^ dm for TPC and 375.03 ± 1.49 mg CAT_eq_ 100 g^−1^ for TFC. The value obtained for TPC and TFC fell within the confidence interval of 95%: 347.16–493.88 mg GAE 100 g^−1^ dm and 269.82–390.86 mg CAT_eq_ 100 g^−1^ dm, respectively. Hence, the results verify the models and confirm that the settings are the best combination to simultaneously maximize TPC and TFC determination in artichoke samples.

An analysis of extraction techniques and conditions applied so far in other studies [18,19,20,21,22,23,24,25,26,27,28,29,30] showed that sonication was applied to phenolic extraction from artichoke samples only in the study by Rouphael et al. [25], who nevertheless used different conditions: room temperature, SSR 10 mL g^−1^, and methanol/water (70:30, *v*/*v*) as extracting mixture. The analysis of the literature also showed that phenolics have mostly been extracted from artichokes through homogenization [18,22,24] or stirring [19,23,26]; ethanol has been also used as extracting solvent; and a wide range of SSR have been applied. For this reason, it is hardly possible to make a comparison of TPC in the different cultivars of artichokes analyzed in the literature. No study has, so far, determined TFC in artichokes. Our study is also the first that has applied RSM to an UAE of phenolics from artichoke heads.

### 3.3. Phenolic Profile at Optimal Extraction Conditions

The phenolic profiles of artichoke extracts obtained at optimal extraction conditions were recorded at 260 nm and 320 nm. These two wavelengths are commonly used in the HPLC analysis of phenolic compounds: the former is the most general detection wavelength used for the simultaneous determination of different phenolic molecules, while the latter is the wavelength used for flavonoid detection. 

**Figure 3:**
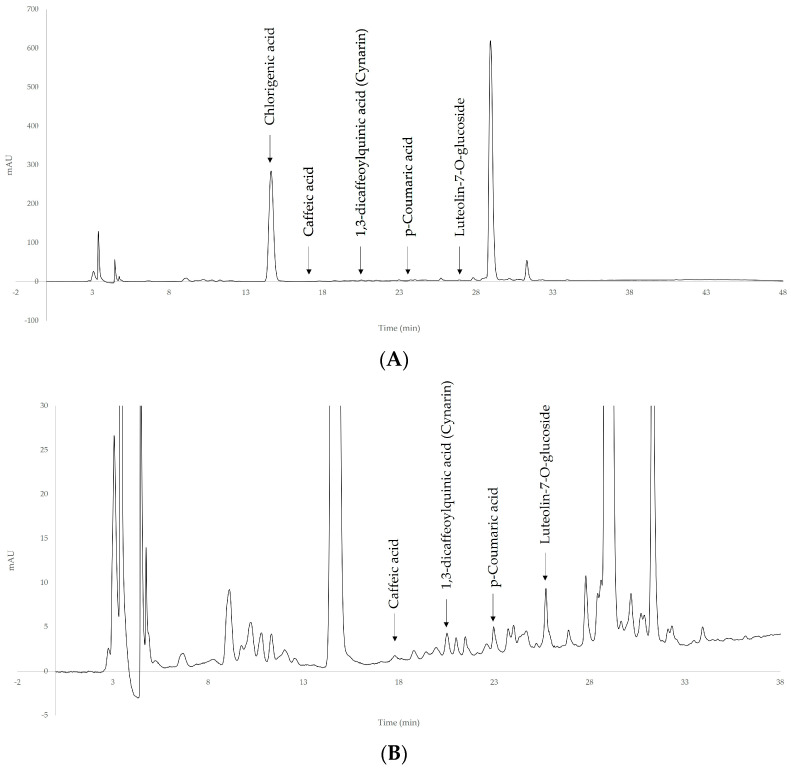
Representative chromatogram of phenolic extracts at 320 nm (**A**) and zoom on peaks of identified compounds (**B**).

The maximum absorbance of target compounds was observed at 320 nm wavelength. The representative chromatogram of artichoke phenolic extract is shown in Figure 3.

Caffeoylquinic acid derivatives were identified in artichoke extracts. They are reported as the major chemical components of globe artichoke [31]. Cynarin, also referred to as 1,3-dicaffeoylquinic acid, was eluted at 20.5 min, and its area was about 0.3% of the total area. Its content was 0.25 mg g^−1^ dm. According to Lattanzio et al., cynarin is the most well-known dicaffeoylquinic acid derivative identified in extracts from artichoke heads and leaves [31]. In addition to cynarin, 1,5-*O*-dicaffeoylquinic acid was tentatively identified at 28.9 min based on its UV spectra and data from the scientific literature [27,28]. This molecule was found to be the main component of artichoke extract. The area of this peak was, in fact, 56% of the total peak area. As regards mono-caffeoylquinic derivatives, chlorogenic acid (5-*O*-caffeoylquinic acid) was found to be the most abundant, which is in keeping with Lattanzio et al. [31], who reported chlorogenic acid is the most important caffeoylquinic acid derivative in artichoke heads and leaves. The content of chlorogenic acid was 24.93 mg g^−1^ dm (Table 5). Chlorogenic acid has been found to be effective in the treatment of metabolic syndrome and related disorders, including diabetes, dyslipidemia, obesity, and hypertension. It has antioxidant activity, mainly against lipid oxidation [32]. 

In plants, caffeoylquinic acid derivatives play a defensive role against biotic and abiotic stresses. In humans, caffeoylquinic acid derivatives have several beneficial effects due to their antioxidant, antibacterial, antiviral, anti-Alzheimer, and neuroprotective activity [33,34,35]. In addition, caffeoylquinic acids have been found to inhibit α-glucosidase and mitigate lifestyle-related diseases, such as diabetes [36]. 

Caffeic and *p*-coumaric acids were also identified in artichoke extracts. They were eluted at 18.0 and 23.8 min, respectively. Both are involved in the biosynthetic pathways of caffeoylquinic acid derivatives [37]. Moreover, caffeic acid may also result from caffeoylquinic acid degradation due to heating. Dicaffeoylquinic acids degrade to the corresponding mono caffeoylquinic acid and then to caffeic and quinic acids, depending on the heating time and temperature [38]. Caffeic acid was also determined in the Romanesco clone C3 but was absent in the Violetto di Provenza and in the Violetto di Sicilia [39], while Negro et al. found this phenolic acid in artichoke samples of the genotypes Blanca Tudela, Mola, Bianco Ostuni, S. Erasmo, Tondo Paestum, and Violetto di Provenza [21], with values ranging from 12.66 to 105.51 mg kg^−1^ of fresh weight (fw). The content was much higher than found in the sample under investigation (0.08 mg g^−1^ dm).

In regard to flavonoids, only the flavone luteolin-7-*O*-glucoside was identified in the methanolic extracts. It shows a C_6_-C_3_-C_6_ structure with two benzene rings and a third, oxygen-containing ring, with a 2-3 carbon double bond. It has been reported to have antioxidant, anti-tumor, anti-inflammatory, and anti-apoptotic activities [40]. The antioxidant activity is higher than apigenin-7-*O*-glycosides and kaempferol 3-*O*-glycosides, since it is a dihydroxy B-ring substituted flavonoid rather than a monohydroxy B-ring-substituted flavonoids [41]. Upon exposure to UV-B, UV-A, or photosynthetic active radiation, the ratio of luteolin to the apigenin derivatives drastically increases. Hence, luteolin derivatives are more abundant in artichoke waste than in the edible parts. In artichoke waste methanolic extracts, luteolin values ranging from 442 to 469 μg g^−1^ have been reported [42]. Values are approximately 10-fold higher than those observed in the sample under investigation.

## 4. Conclusions

The study allowed the optimization of the extraction of phenolic acids and flavonoids in artichoke samples by considering the combined effect of ultrasounds, extraction time, temperature, and solvent-to-sample ratio. The optimal extraction conditions were 20 min, 60 °C, and 50 mL g^−1^ SSR. Hence, an analytical procedure based on low solvent volumes, short time, and low energy was applied. 

The HPLC analysis of the optimized extracts enabled the accurate quantification of phenolic compounds. Chlorogenic acid was the most abundant component identified and quantified in artichoke extract, followed by cynarin, the most well-known dicaffeoylquinic acid derivative. A small amount of caffeic acid, *p*-coumaric acid and luteolin-7-*O* glucoside was also detected. Further components present in the extract might be identified in future studies, depending on the availability of additional pure standard phenolic compounds.

## Figures and Tables

**Table 1:** Factors and levels used in the Box–Behnken design matrix.

Factor	Symbol	Levels		
		Low (−1)	Intermediate (0)	High (+1)
Temperature (°C)	X_2_	20	40	60
Time (min)	X_1_	10	20	30
Solvent-to-sample ratio (SSR; mL g^−1^)	X_3_	20	35	50

**Table 2:** Chromatographic parameters of phenolic compounds determined by HPLC (LOD: limit of detection; LOQ: limit of quantification).

Phenolic Compounds	Regression Equation	R^2^	LOD (μg mL^−1^)	LOD (μg mL^−1^)
Chlorogenic acid	y = 1.1634x + 8.6200	0.9968	24.21	73.36
Caffeic acid	y = 2.3381x − 0.0892	0.9995	1.85	5.62
1,3-dicaffeoylquinic acid	y = 1.4314x − 0.9832	0.9997	2.11	6.38
*p*-coumaric acid	y = 2.4001x − 1.9857	0.9988	2.11	6.02
Luteolin-7-*O*-glucoside	y = 0.3803x + 0.1394	0.9985	8.01	24.27

**Table 3:** Box–Behnken design for the UAE of phenolic compounds from artichoke heads: run conditions and responses.

Run Order	Independent Variables Actual Values			Responses	
	Temperature (X_1_)	Time (X_2_)	SSR (X_3_)	TPC	TFC
	(°C)	(min)	(mL g^−1^)	(mg GAE 100 g^−1^ dm)	(mg CAT_eq_ 100 g^−1^ dm)
#1	20	20	20	393.06 ± 1.39	296.00 ± 26.35
#2	20	10	35	347.16 ± 15.28	333.42 ± 1.91
#3	40	20	35	385.11 ± 9.69	324.47 ± 17.50
#4	40	10	50	493.88 ± 14.12	360.64 ± 6.28
#5	20	20	50	390.85 ± 2.74	324.92 ± 13.62
#6	40	10	20	391.81 ± 17.45	274.70 ± 22.02
#7	60	10	35	433.86 ± 6.63	343.75 ± 25.57
#8	20	30	35	357.98 ± 3.58	329.69 ± 2.10
#9	40	30	20	365.67 ± 2.38	269.82 ± 15.98
#10	40	30	50	447.79 ± 1.80	372.75 ± 14.96
#11	40	20	35	412.71 ± 12.00	294.44 ± 18.51
#12	60	20	20	364.47 ± 4.77	311.51 ± 8.17
#13	60	30	35	441.27 ± 10.27	327.53 ± 7.47
#14	40	20	35	420.25 ± 9.71	297.02 ± 15.45
#15	60	20	50	485.09 ± 1.82	390.86 ± 20.78

SSR: Solvent-to-sample ratio; TPC: total phenolic content; TFC: total flavonoid content; GAE: gallic acid equivalent; CAT_equ_: catechin equivalent.

**Table 4:** Analysis of variance (ANOVA) of the models for TPC and TFC.

	Source	SS	df	MS	F Value	*p*-Value
TPC model						
	Regression	22,158.11	3	7386.04	13.83	0.0005
	X_1_	6941.01	1	6941.01	12.99	0.0041
	X_3_	11,445.54	1	11,445.54	21.42	0.0007
	X_1_X_3_	3771.56	1	3771.56	7.06	0.0223
	Residuals	5876.52	11	534.23		
	Lack-of-Fit	5192.07	9	576.90	1.69	0.4272
	Pure Error	684.45	2	342.22		
	Total	28,034.63	14			
	R^2^ = 79.04%					
	adj. R^2^ = 73.32%					
TFC model						
	Regression	13,361.19	3	4453.73	15.04	0.0003
	X_1_	1003.86	1	1003.86	3.39	0.0927
	X_3_	11,037.27	1	11,037.27	37.28	<0.0001
	X_1_^2^	1320.07	1	1320.07	4.46	0.0584
	Residuals	3256.34	11	296.03		
	Lack-of-Fit	2702.46	9	300.27	1.08	0.5678
	Pure Error	553.88	2	276.94		
	Total	16,617.53	14			
	R^2^ = 80.40%					
	adj. R^2^ = 75.06%					

SS: sum of squares; df: degree of freedom; MS: mean square; F: variance; P: tests statistics.

**Table 5:** Phenolic compounds were identified and quantified in artichoke extracts.

Phenolic Compound	Class	Retention Time	Concentration
		(min)	(mg g^−1^ dm)
Chlorogenic acid	Caffeoylquinic acid derivative	14.5	24.93 ± 0.28
Caffeic acid	Phenolic acid	17.7	0.08 ± 0.00
1,3-Dicaffeoylquinic acid	Caffeoylquinic acid derivative	20.5	0.25 ± 0.00
*p*-Coumaric acid	Phenolic acid	23.7	0.12 ± 0.00
Luteolin-7-*O*-glucoside	Flavonoid	25.6	0.04 ± 0.00

## Data Availability

The data presented in this study are available on request from the corresponding author.
